# Special Issue: “Molecular and Cellular Mechanisms of Retinal Diseases”

**DOI:** 10.3390/ijms27146132

**Published:** 2026-07-09

**Authors:** Maria Consiglia Trotta

**Affiliations:** Department of Experimental Medicine, University of Campania “Luigi Vanvitelli”, 80138 Naples, Italy; mariaconsiglia.trotta2@unicampania.it

Retinal diseases are among the leading causes of irreversible visual impairment worldwide and represent a growing public health challenge due to population aging and the increasing prevalence of metabolic disorders. Despite major advances in retinal imaging, pharmacological treatments, and surgical techniques, conditions such as diabetic retinopathy (DR), retinal vein occlusion (RVO), and age-related macular degeneration (AMD) often continue to progress (Contributions 1–5).

Retinal pathologies result from complex interactions among multiple biological processes, including oxidative stress, inflammation, hypoxia, mitochondrial dysfunction, metabolic imbalance, endothelial barrier breakdown, neurodegeneration, and pathological angiogenesis. Although vascular endothelial growth factor (VEGF) is a central mediator of retinal angiogenesis, anti-VEGF therapies are often insufficient and require repeated intravitreal injections.

In the search for additional disease-specific angiogenic factors, Huang et al. evaluated humanized antibodies targeting Secretogranin III (Scg3), an angiogenic factor that selectively promotes pathological angiogenesis and vascular leakage in retinal disease conditions. Their study compared four anti-Scg3 antibodies and showed that affinity-matured hFabs (particularly EBP3) displayed markedly improved binding, strong inhibition of endothelial proliferation and migration in vitro, and superior therapeutic efficacy in vivo for reducing DR-related vascular leakage and neovascularization, outperforming both EBP2 and aflibercept in paired mouse models (Contribution 1). Importantly, these results suggest that targeting disease-selective angiogenic pathways may provide more specific approaches to controlling retinal vascular dysfunction. 

To further elucidate the mechanisms regulating this process, further studies investigated the role of nitric oxide (NO) signaling in retinal vascular responses. The contribution by Warden et al. demonstrates that the combination of citrulline and arginine promoted angiogenic responses and increased endothelial permeability in human retinal microvascular endothelial cells through the activation of the endothelial nitric oxide synthase (eNOS)/NO pathway. This was evidenced by increased eNOS expression, phosphorylation, and NO production, leading to enhanced proliferation, migration, and tube formation. Conversely, the inhibition of eNOS abolished these effects, confirming a central role for NO signaling in regulating endothelial dysfunction, without affecting arginase activity (Contribution 2).

NO signaling has also emerged as a key mediator in the regulation of hypoxia-induced VEGF production. Studies using retinal endothelial and Müller cells have previously identified β3-adrenoceptor signaling as a critical regulator of this process, promoting VEGF expression through a NO-dependent mechanism. Lucchesi et al. showed that β3-adrenergic receptor signaling regulates NOS activity in retinal endothelial and Müller cells, modulating VEGF expression independently of hypoxia-inducible factor 1 alpha (HIF-1α) stabilization. Their findings indicate a dual mechanism in which hypoxia-driven HIF-1α signaling and β3-adrenergic–dependent NO production converge to control VEGF levels, identifying nitric oxide as a key integrator of metabolic and hypoxic pathways and a potential therapeutic target in retinal angiogenesis (Contribution 3).

Beyond vascular abnormalities, emerging evidence indicates that retinal degeneration is strongly influenced by oxidative stress, impaired cellular homeostasis and loss of intrinsic protective mechanisms. The study by Lu et al., investigating adeno-associated virus (AAV)-mediated BMI1 gene delivery, is relevant in the context of oxidative stress-driven retinal degeneration. BMI1 gene therapy targeting pathways involved in DNA repair, mitochondrial function, and oxidative stress response enhanced retinal homeostasis. In particular, suprachoroidal delivery achieved efficient transgene expression with the preservation of retinal structure and function after oxidative injury, suggesting that boosting endogenous protective mechanisms may provide sustained protection against degeneration. This suggests minimally invasive gene therapy approaches as a promising strategy for dry AMD (Contribution 4).

The translation of molecular discoveries into clinical practice also relies on the identification and validation of reliable biomarkers capable of improving patient stratification and predicting therapeutic response. As retinal therapies become increasingly mechanism-driven, the integration of such biomarkers into clinical workflows may represent a crucial step toward advancing precision medicine in retinal disorders. While retinal imaging has provided several structural indicators associated with visual prognosis in RVO, the search for complementary systemic biomarkers remains at an early stage. The pilot study by Gesualdo et al. suggests that circulating xanthine oxidase (XO) and thrombospondin-1 (TSP-1), markers linked to oxidative stress and angiogenesis, may have prognostic value in retinal vein occlusion, as lower levels are associated with better visual outcomes after dexamethasone treatment. This evidence supports their potential use alongside imaging for improved patient stratification and personalized therapy, pending validation in larger cohorts (Contribution 5).

Overall, the studies in this Special Issue highlight the complex and interconnected molecular mechanisms underlying retinal diseases. Advances in molecular biology are expanding therapeutic targets beyond VEGF, enabling more disease-specific approaches with the potential to improve efficacy and reduce treatment burden. In parallel, progress in antibody engineering, gene therapy, and biomarker discovery is driving a shift toward increasingly personalized retinal medicine. Taken together, these developments reflect a rapidly evolving field in which basic and translational research are progressively converging to address the persistent unmet clinical needs in retinal disorders.

## Abbreviations

The following abbreviations are used in this manuscript:

AMD	Age-related Macular Degeneration
AAV	Adeno-associated virus
DR	Diabetic Retinopathy
eNOS	endothelial Nitric Oxide Synthase
HIF-1α	Hypoxia-inducible factor 1 alpha
NO	Nitric Oxide
RVO	Retinal Vein Occlusion
Scg3	Secretogranin III
TSP-1	Thrombospondin-1
VEGF	Vascular Endothelial Growth Factor
XO	Xanthine Oxidase

